# Anaplastic thyroid carcinoma with osteoclast-like giant cells: a case report and a study of a potential therapeutic approach

**DOI:** 10.1007/s00795-025-00443-1

**Published:** 2025-07-24

**Authors:** Kaori Yukino, Yoshihiro Komohara, Shukang Zhao, Rin Yamada, Yukio Fujiwara, Akira Murakami, Yu Shimoda, Haruki Saito, Yorihisa Orita

**Affiliations:** 1 Department of Otolaryngology-Head and Neck Surgery, Kumamoto University Graduate School of Medicine, Kumamoto, Japan; 2 Department of Cell Pathology, Graduate School of Medical Sciences, Kumamoto University, 1-1-1 Honjo, Chuo-Ku, Kumamoto, 8608556 Japan

**Keywords:** Thyroid cancer, Anaplastic thyroid carcinoma, Osteoclastic giant cells, PD-L1, TGF-β

## Abstract

Anaplastic thyroid carcinoma (ATC) is a highly aggressive neoplasm with no effective treatment options. ATC with osteoclast-like giant cells (OGCs; ATC/OGC) is a rare variant of ATC, and no detailed pathological examination has been reported to date. A 59-year-old woman presented with sudden neck swelling. Computed tomography revealed a 5 cm tumor in the thyroid gland, which was surgically resected. Pathological examination revealed a diagnosis of ATC/OGC. The patient succumbed to progressive lung metastases within four months despite postoperative lenvatinib therapy. Immunohistochemical (IHC) examination indicated absence of PD-L1 expression in the OGCs, which comprised the majority of the tumor, with only sparse T cell infiltration in the area occupied by OGCs. Increased TGF-β expression was observed in the area containing OGCs, and both OGCs and infiltrating myeloid cells, including CD1a/CD11c-positive dendritic cells and CD68/CD163/CD204-positive macrophages, appeared to produce TGF-β. Pathological analysis of this case suggests that OGCs might be involved in immune suppression by secreting TGF-β, potentially serving as a critical cytokine in the immunosuppressive microenvironment of ATC/OGC. TGF-β-targeted therapy might be useful in the treatment of this very rare subtype of ATC.

**Supplementary Information:**

The online version contains supplementary material available at 10.1007/s00795-025-00443-1.

## Introduction

Traditionally, primary surgical treatment followed by adjuvant chemo-radiotherapy or chemotherapeutic treatment using anti-cancer drugs, such as doxorubicin, cisplatin, or paclitaxel, has been used for anaplastic thyroid carcinoma (ATC), which has a median survival of approximately 5 months and a one-year overall survival rate of 20% [1–4]. Recently, targeted therapy has become available for ATC patients who demonstrate BRAF V600E mutation or certain gene fusions, leading to improved survival rates [5, 6]. Combination therapy with a programmed death-1 (PD-1) inhibitor along with various kinds of kinase inhibitors has also emerged as an effective approach in the treatment of ATC [7], and a remarkable effect of even ICIs alone has been shown in some ATC patients in previous studies and case reports [4, 8, 9]. However, there are various ATC subtypes, each of which might respond differently to treatment. Given the aggressive progression of ATC, rapidly determining and initiating the most effective therapy is extremely challenging. Here, we present a rare subtype of ATC with osteoclast-like giant cells (OGCs; ATC/OGC) which is also known as osteoclastic variant of ATC. To the best of our knowledge, fewer than 20 cases of ATC/OGC have been reported in the English literature, and only seven case reports are available in the PubMed database at the time of this writing (Table 1 [10–15]). Although there is no clear pathological definition of ATC/OGC, the presence of CD68-positive OGCs has been considered a key finding for diagnosing ATC/OGC based on previous studies [10, 13–15]. In the present case, we also examined the expressions of immunosuppressive molecules and myeloid cell markers in OGCs by means of immunohistochemical (IHC) analysis.

**Table 1:** Case reports of ATC/OGCs previously published in PubMed, including the current case

	Age (y)	Sex	Size	Survival time	Other findings	References
1)	81	Female	6.7 cm	–	OGCs were positive for CD68 and PD-L1 (CPS: 20%)	[10]
2)	90	Male	12 cm	6 months	No findings	[11]
3)	50	Male	7 cm	–	Fine needle aspiration cytology demonstrated OGCs	[12]
4)	50	Male	6 cm	–	OGCs were positive for CD68 and cathepsin K	[13]
5)	67	Male	13 cm	–	OGCs were positive for CD68	[14]
6)	48	Male	12.8 cm	1 month	OGCs were positive for CD68	[15]
7)	68	Male	13.5 cm	3 months	No findings	[15]
8)	59	Female	7.0 cm	4 months	OGCs were positive for CD68	Present case

*ATC* anaplastic thyroid carcinoma, *OGCs* osteoclast-like giant cells, *ATC/OGCs* ATC with osteoclast-like giant cells, *PD-L1* programmed cell death ligand 1, *CPS* combined positive score

## Case report

**Fig. 1:**
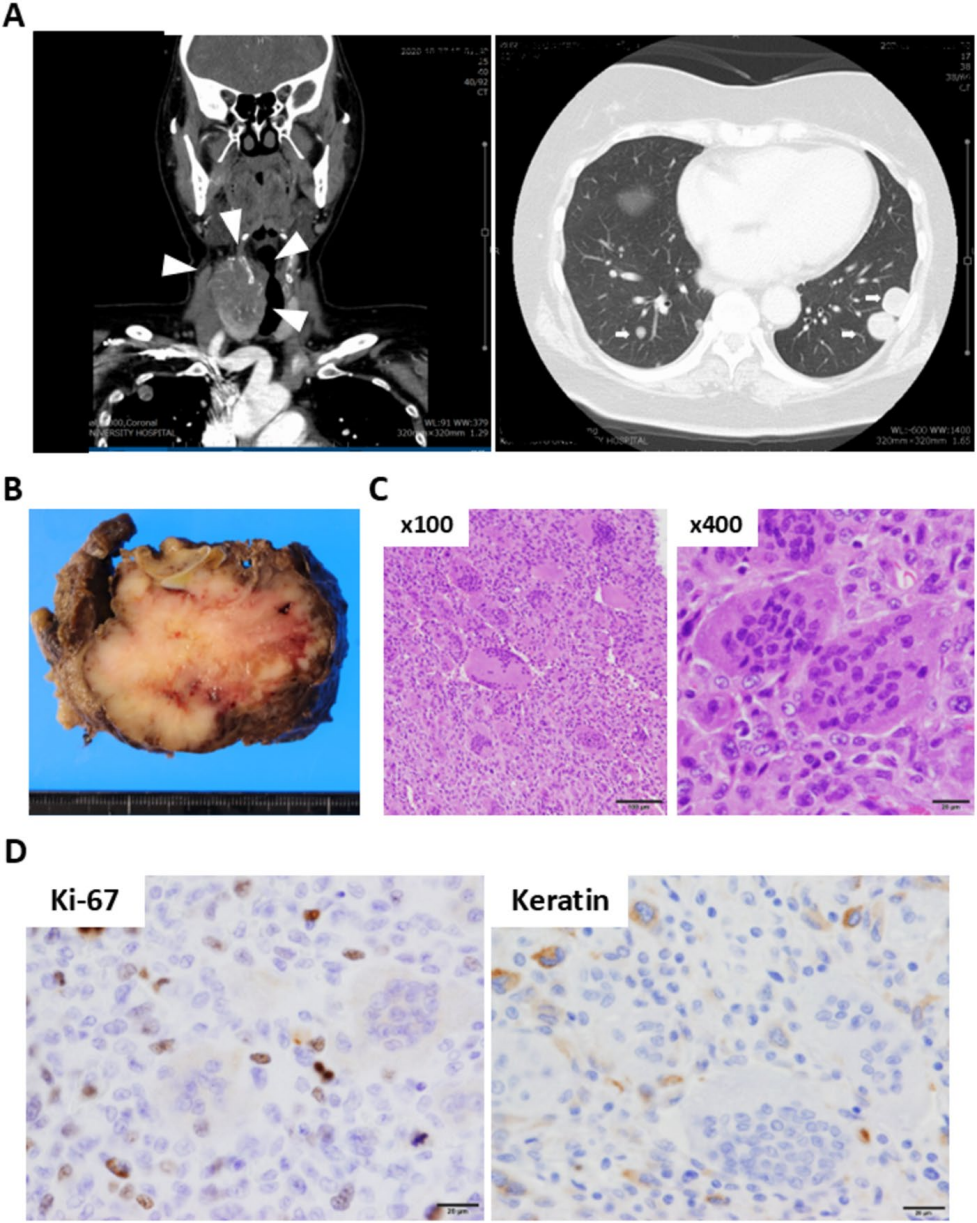
Routine radiological and pathological examination of the resected specimen. **A** Contrast-enhanced computed tomography (CT) showed a tumor approximately 5 cm in size in the anterior neck. Multiple metastases were observed in both lungs. **B** A section of the right lobe of the thyroid gland was entirely replaced by the tumor. **C** Microscopic examination revealed a poorly differentiated tumor with prominent osteoclast-like giant cells (OGCs). **D** IHC was performed using anti-Ki-67 antibody (clone MIB1, Leica Biosystems, Nussloch, Germany) and anti-Keratin antibody (clone OSCAR, BioLegend, San Diego, CA, USA). Immunohistochemical (IHC) analysis showed that Ki-67 and keratin were negative in OGCs, but positive in undifferentiated spindle cells

**Fig. 2:**
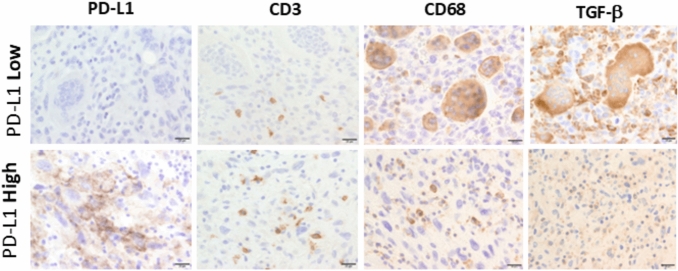
Additional immunohistochemical (IHC) studies in the tumor tissue. IHC evaluation for PD-L1 (clone 22C3, Leica), CD3 (clone SP7, Nichirem Tokyo, Japan), CD68 (clone PG-M1, Leica) and TGF-β (clone EPR21143, Abcam, Cambridge, UK) was performed. PD-L1 expression was heterogeneous, with positive expression observed in cancerous areas devoid of osteoclast-like giant cells (OGCs), whereas areas containing OGCs were PD-L1-negative

A 59-year-old Japanese woman initially presented with a 1-month history of a progressively enlarging mass on the right side of the neck, with complaints of dysphagia and dyspnea. Her past medical history was significant for hypertension and diabetes mellitus. Physical examination revealed an approximately 5 cm long, hard, nodular mass on the right side of the neck, that moved with deglutition. The overlying skin appeared normal. Computed tomography (CT) demonstrated a large, heterogeneous, multi-lobulated right thyroid tumor (5.2 × 4.0 × 5.4 cm) invading hypopharynx, larynx, and right internal jugular vein, with tumor thrombus formation (Fig. 1A). Multiple lung metastases were also present in both lung fields (Fig. 1A). Total thyroidectomy and construction of a laryngocutaneous fistula with resection of the muscular layer of the hypopharynx and esophagus were performed to prevent asphyxiation and dysphagia, and to facilitate subsequent therapy. On gross examination, the cut surface of the mass measured 7.0 × 5.0 cm, and it was multinodular, solid and yellowish white in color, with areas of hemorrhage (Fig. 1). Histologic examination showed a poorly differentiated tumor with prominent OGCs (Fig. 1C). IHC studies revealed undifferentiated spindle cells were positive for keratin (Fig. 1D), but negative for PAX8 and TTF1 (not shown). Ki-67 was negative in OGCs, but positive in over 80% of undifferentiated spindle cells based on keratin-positive cell density (Fig. 1D). The OGCs were positive for CD68 (Fig. 2). Histological patterns of papillary and follicular components were not present. Based on these findings, the tumor was diagnosed as ATC/OGC. Subsequently, due to the progressive development of lung metastases postoperatively, lenvatinib therapy was initiated. However, the patient succumbed to her illness within 4 months of diagnosis.

## Additional pathological examinations

**Fig. 3:**
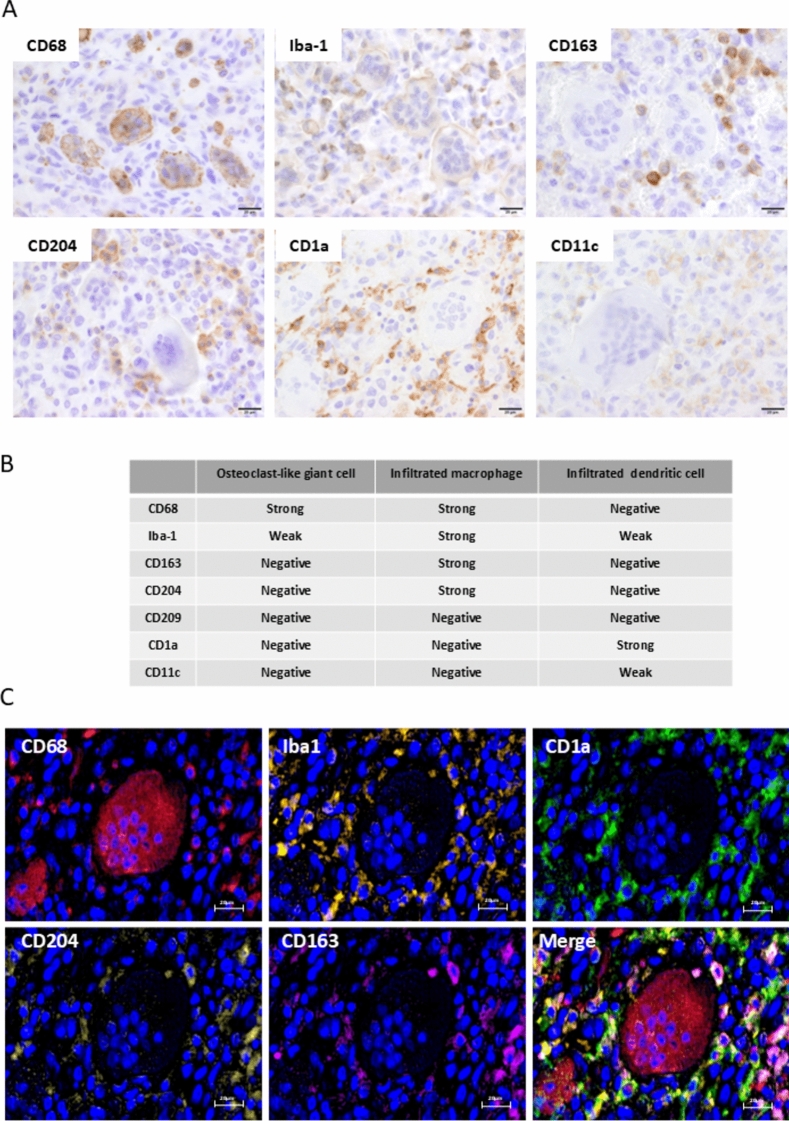
Detailed characterization of osteoclast-like giant cells (OGCs). **A** CD68-positive OGCs exhibited weak positivity for Iba-1, but were negative for macrophage markers (CD163, CD204) and dendritic cell markers (CD1a, CD11c). IHC was performed using anti-CD163 antibody (clone 10D6, Leica), anti-CD204 antibody (clone SR-E5, CosmoBio, Tokyo, Japan), anti-CD1a antibody (clone NCL-L-CD1a, Leica), and anti-CD11c antibody (clone EP1347Y, Abcam). **B** Summary of the IHC results. **C** Multiplex IHC confirmed these findings, revealing that Iba-1-positive myeloid cells could be categorized into CD68/CD163/CD204-positive macrophages and CD1a-positive dendritic cells

IHC studies were performed postmortem to test immune cell-related molecules, such as programmed cell death ligand 1 (PD-L1) and immune cell infiltration using formalin-fixed paraffin-embedded sections of the tumor. Analysis demonstrated heterogeneous PD-L1 expression, with positive expression observed in cancerous areas devoid of OGCs, whereas areas containing OGCs were PD-L1-negative (Fig. 2). OGCs were absent in 5% of the cancerous lesion. Increased infiltration of CD3-positive T cells was detected in PD-L1-positive cancer areas. OGCs and infiltrating macrophages expressed CD68, with higher expression levels in OGCs than in macrophages. Increased transforming growth factor beta (TGF-β) expression was observed in areas containing OGCs, with strong positive signals detected in stromal cells, including OGCs (Fig. 2). CD68-positive OGCs exhibited weak positivity for Iba-1, but were negative for other macrophage markers (CD163, CD204) and dendritic cell markers (CD1a, CD11c) (Fig. 3A, B). Multiplex IHC studies confirmed these findings, revealing that Iba-1-positive myeloid cells could be categorized into OGCs, CD163/CD204-positive macrophages, and CD1a-positive dendritic cells (Fig. 3C, Supporting Fig. 1).

## Discussion

A total of only 7 ATC/OGC (or osteoclastic variant of ATC) cases are present in the PubMed database, as summarized in Table 1 [10–15]. A previous report described multinucleated giant cells were seen in 20 (66%) of 30 ATC cases, while only one case (3%) was diagnosed as osteoclastic variant of ATC with OGCs [16]. Additionally, spindle cells were positive for keratin and vimentin, whereas OGCs were negative for these markers [16]. In other reports, OGCs were characterized as myeloid lineage cells based on IHC analysis since they express myeloid cell markers, such as lysozymes and CD68 [10, 17]. In the present case, OGCs exhibited strong CD68 expression and low Iba-1 expression, but were negative for other macrophage and dendritic cell markers. These findings suggest that CD68, widely used as a macrophage marker, is mostly useful for identifying OGCs.

A previous study reported that PD-L1 expression was present in 65% of ATC cases and absent in 35% [18]. Several studies have investigated PD-L1 expression in ATCs, with variable findings, as summarized in Table 2 [8, 19–24]. According to Monikongkona et al. [19], clinical trials targeting PD-1/PD-L1 immune checkpoints demonstrated limited efficacy against ATC. A literature analysis revealed that only 12.1% of PD-L1-positive ATC patients achieved a complete response to anti-PD therapy, with partial response, stable disease and progressive disease being observed in 19%, 15.5% and 53.4% of cases, respectively [19]. Sarcomatoid ATC is more likely to be PD-L1-negative compared to epithelioid and pleomorphic ATC [20]. In a case report of ATC [8], PD-L1 expression was detected in 30% of tumor cells, and combination therapy with an anti-PD-1 antibody and nab-paclitaxel induced partial remission. Thus, targeted therapy against the PD-1/PD-L1 signaling pathway might be effective in some PD-L1-positive ATC cases. However, in the present case, the majority of the tumor area was occupied with OGCs that were PD-L1 negative, along with limited T cell infiltration. Although PD-L1 expression is reportedly associated with poor survival outcomes in ATC [21], it is not considered to affect the prognosis of ATC/OGC, suggesting that OGCs might contribute to an as yet unknown immunosuppressive mechanism.

**Table 2:** Articles that described PD-L1 expression in ATCs

	Number of cases	Sex (male/female)	ICI therapy	Comments on PD-L1	PMID
1)	1	1/0	Yes	The tumor proportion score (TPS) of PD-L1 was 30%. Combination therapy of anti-PD-1 antibody and nab-paclitaxel induced partial remission, with reduction in the size of the thyroid tumor and liver metastases	[8]
2)	13	6/7	No	ATC tumors showed high PD-L1 expression, with a positivity rate of 69%. High PD-L1 expression was associated with lower progression-free survival	[21]
3)	3	No information	No	PD-L1 was positive in all three ATC cases	[22]
4)	26	8/18	No	ATC tissue samples from 17 patients (65.4%) were PD-L1-positive	[19]
5)	15	6/9	No	PD-L1 TPS with a 1% cut-off was detected in 9/15 (60%) ATC cases. PD-L1 expression was detected in immune cells in 10/15 (73%) ATC cases	[23]
6)	16	11/5	No	PD-L1 expression in tumor cells and immune cells was seen in 13 of 16 cases and in 7 of 16 patients, respectively. PD-L1 positivity in immune cells was associated with shortened survival time	[24]
7)	179	56/123	No	At a cut-off TPS of 1%, 73.2% of ATC cases (131 of 179) were PD-L1-positive. The overall survival of patients did not differ significantly based on PD-L1 expression status	[20]

In a previous study of single-cell RNA sequencing analysis of ATC samples, ATC cases were categorized as inflammatory and mesenchymal types, with identification of TGF-β-gene overexpression in mesenchymal ATC [25]. TGF-β is well-known for its role in tumor invasion, by promoting epithelial-mesenchymal transition, fibrosis and immune suppression [26, 27]. Recently, an inhibitor targeting TGF-β has been under development for use in anti-cancer immunotherapy [28]. In the present case, TGF-β expression was investigated in the tumor area that contained OGCs, which formed the bulk of the tumor, and which had a lower density of lymphocytes and were classified as immunologically “COLD” areas in contrast to PD-L1-positive regions, which were classified as “HOT” areas. This suggests that TGF-β might be a key immunosuppressive factor contributing to immune evasion by OGCs. 

In conclusion, we present a case of ATC/OGC. The majority of the tumor was occupied by OGCs that did not express PD-L1, with limited T cell infiltration, but which strongly expressed TGF-β. Hence, targeting TGF-β therapy might be useful in the treatment of this very rare subtype of ATC.

## Supplementary Information

Below is the link to the electronic supplementary material.

Supplementary file1 (TIF 525 KB)
