# Supplementary figures and images for: Anaplastic thyroid carcinoma with osteoclast-like giant cells: a case report and a study of a potential therapeutic approach
Source: Med Mol Morphol. 2025 Jul 24;59(1):64–70. doi: 10.1007/s00795-025-00443-1 (PMC12967400; doi:10.1007/s00795-025-00443-1)

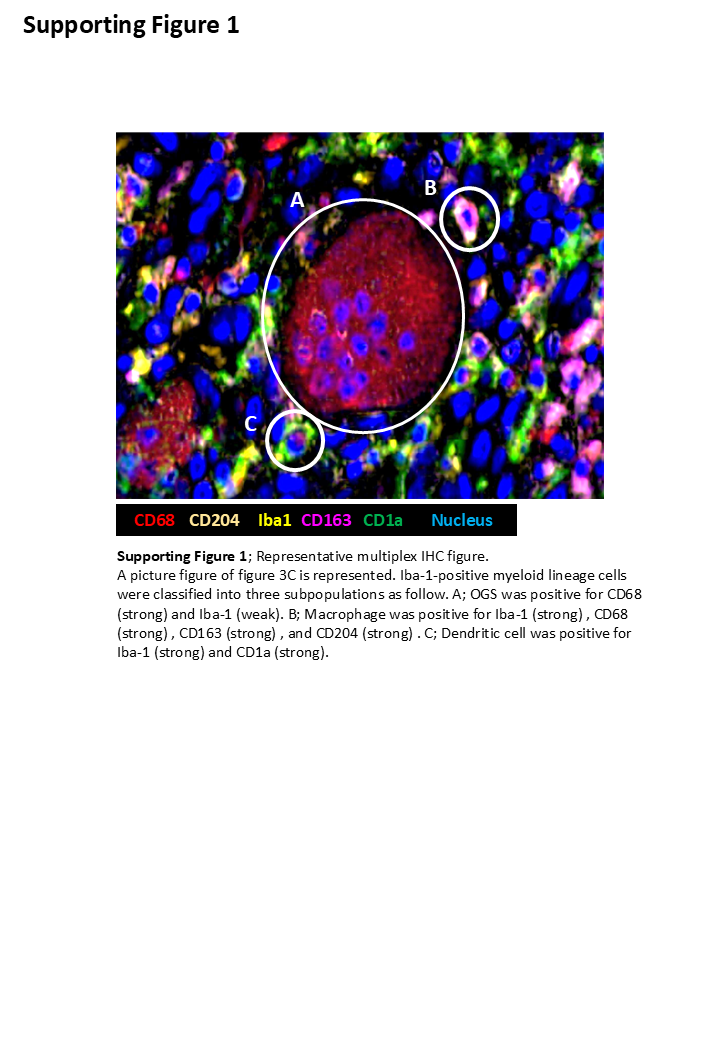

Supplement: Supplementary file 1 — Supplementary file1 (TIF 525 KB) [file 795_2025_443_MOESM1_ESM.tif]
